# *Rosa platyacantha* Schrenk from Kazakhstan—Natural Source of Bioactive Compounds with Cosmetic Significance

**DOI:** 10.3390/molecules26092578

**Published:** 2021-04-28

**Authors:** Askhat Sabitov, Katarzyna Gaweł-Bęben, Zuriyadda Sakipova, Marcelina Strzępek-Gomółka, Uliana Hoian, Elmira Satbayeva, Kazimierz Głowniak, Agnieszka Ludwiczuk

**Affiliations:** 1 Department of Pharmaceutical Technology, Asfendiyarov Kazakh National Medical University, 94 Tole bi Str., Almaty 050012, Kazakhstan; acxam78@gmail.com (A.S.); sakipova.z@kaznmu.kz (Z.S.); satbaeva.e@kaznmu.kz (E.S.); 2 Department of Cosmetology, The University of Information Technology and Management in Rzeszow, Sucharskiego 2, 35-225 Rzeszów, Poland; mstrzepek@wsiz.edu.pl (M.S.-G.); uhoian@wsiz.edu.pl (U.H.); kglowniak@wsiz.edu.pl (K.G.); 3 Independent Laboratory of Natural Products Chemistry, Department of Pharmacognosy, Medical University of Lublin, 1 Chodzki Str., 20-093 Lublin, Poland; aludwiczuk@pharmacognosy.org

**Keywords:** *Rosa platyacantha*, HPLC/ESI-QTOF-MS, antioxidant, tyrosinase, elastase, collagenase melanoma, in vitro cytotoxicity

## Abstract

Plants belonging to the *Rosa* genus are known for their high content of bioactive molecules and broad spectrum of healing and cosmetic activities. *Rosa platyacantha* Schrenk is a wild-type species abundant in the mountainous regions of Kazakhstan. The phytochemical composition as well as the bioactivity of *R. platyacantha* extracts have not been fully investigated to date. In this study, various parts of *R. platyacantha* plant, collected in Almaty region, Kazakhstan, were used to prepare five hydroalcoholic extracts (R1–R5). The extracts were compared for the content of phytochemicals and selected biological activities, which are important for the potential cosmetic application of *R. platyacantha*. Extract R3, prepared from flower buds, showed the most significant antioxidant and tyrosinase inhibitory potential, decreasing the monophenolase and diphenolase activities of tyrosinase. Extract R3 showed also collagenase inhibitory activity and cytotoxicity against human melanoma cells A375, being less cytotoxic for noncancerous skin keratinocytes HaCaT. Analysis of fractions E and F, obtained from R3 extracts, revealed that quercetin, kaempferol, rutin, and their derivatives are more likely responsible for the tyrosinase inhibitory properties of *R. platyacantha* extracts.

## 1. Introduction

Species belonging to genus *Rosa* are among one the most popular ornamental and cutter plants on the planet. Genus *Rosa* is composed of over 200 species; however, only around 10–15 species were used to propagate modern cultivars, making the remaining wild-type species less studied [1]. Extracts and compounds isolated from various *Rosa* species are widely used as traditional medicines [2,3] and explored against a variety of diseases such as skin disorders [4], hepatotoxicity [5], renal disturbances [6], diarrhea [7], arthritis [8], diabetes [9], hyperlipidaemia [10], and cancer [11]. Alcoholic extracts from different *Rosa* species have shown also some antiviral activity with no cytotoxic effects [12]. The anticancer effect of *Rosa* is explained by the rich content of antioxidants. It was shown that neutral and acidic phenols are the main components of the extract which has antiproliferative and apoptotic effect on the cancer cells [13]. *Rosa* extracts also could have an opposite effect and increase the cell survival; for example, extracts of *R. canina* contain the isoflavone phytoestrogens, which increase the survival of estrogen-dependent cancer cell lines such as breast cancer cells (MCF-7) in vitro [14]. Extracts from other *Rosa* sp., such as *R. rugosa*, contain also other active compounds affecting the epigenetics of cancer cells, inhibiting the activity of histone acetyltransferase and inducing apoptosis of prostate cancer cell lines [15].

*Rosa* species are also a particularly rich source of active ingredients for the cosmetic industry. *R. alba, R. borboniana, R. canina, R. centifolia, R. damascena, R. davurica, R. floribunda, R. gallica, R. hybrida, R. moschata, R. multiflora, R. rubiginosa, R. rugosa*, and *R. spinosissima* are among the species currently used in cosmetic products, possessing scientifically proven skin care activities [16]. For example, ethanolic extract from *R. multiflora* flowers prevents ultraviolet (UV)-induced biochemical damages, leading to photoaging by decreasing reactive oxygen species (ROS), interleukin (IL)-6, IL-8, and matrix metalloproteinase (MMP)-1 levels [17]. *R. canina* hip powder, containing seeds and shells, was recently shown to increase cell longevity, improve skin wrinkles, moisture, and elasticity [4]. Petal extract of *R. gallica* decreased the expression of solar UV-induced MMP-1, which is a hallmark of wrinkle formation [18]. Extracts and compounds isolated from *R. canina, R. gallica*, and *R. rugosa* have been extensively studied to assess their efficacy as potential skin-lightening ingredients [19,20,21]. It was found that polyphenols abundant in *Rosa* sp. extracts, especially quercetin, kaempferol, and ellagic acid possess in vitro inhibitory activity toward tyrosinase, which is an enzyme responsible for melanin synthesis [21].

The phytochemical composition of *Rosa* genus varies between different and the same species and depends on geographic locations, ecology, soil composition, and other environmental factors, especially for the concentration of vitamin C and phenolic compounds [22]. Recent studies by Dani et al. showed that the phytochemical composition of *Rosa* sp. depends also on the floral development and senescence [23]. The most studied parts of *Rosa* plants are rose hips, which are known as a rich source of natural antioxidants, e.g., PUFA (polyunsaturated fatty acids) such as linoleic acid, but also flavonoids, triterpenoids, and phytosterols [24]. Galactolipids, which are also found in rose hips, have shown some anti-inflammatory and antitumor activities [25]. Flower buds of *R. rugosa* were shown recently to contain acidic polysaccharides with antioxidant and anti-aging properties [26] as well as neuroactive depside glucosides, flavonoids, and tannins [27]. Flavonoids, including kaempferol and quercetin derivatives, were also found in *R. damascena* flower buds [28]. The leaves of various *Rosa* species were shown to contain significant amounts of polyphenols, comprising from to 5.7 ± 0.08% to 15.2 ± 0.21% of dried weight [29]. In *R. canina*, the polyphenolic content is higher in the leaves than in the fruits [30].

In the current research, we have studied the phytochemical composition and selected cosmetic activities of *Rosa platyacantha* Schrenk growing in the mountains of Trans-Ili Alatau, Northern Tian Shan Mountain (Almaty, Kazakhstan). This *Rosa* species has not been characterized in the scientific literature to date. In addition, it is evident that plants growing in unfavorable environmental conditions, such as the mountains of Almaty region, are generally rich in phytochemical compounds with a broad spectrum of biological activities, including antioxidant, anti-inflammatory, and UV-protecting properties [21,31,32,33]. The extracts from such plants are a potentially interesting source of active ingredients for cosmetic products protecting the skin from the harmful impact of environmental factors, such as air pollution and UV radiation. For that reason, in this study, *R. platyacantha* extracts prepared from various parts of the plant were investigated for their antioxidant potential, elastase, collagenase, and tyrosinase inhibitory properties, and anti-melanoma activity in vitro. The extract from flower buds, with the most significant tyrosinase inhibitory properties, was fractionated in order to identify the compounds responsible for the tyrosinase inhibition.

## 2. Results and Discussion

### 2.1. Phytochemical Content and Antioxidant Extracts from Various Parts of R. platyacantha

Hydroalcoholic extracts, prepared from the flowers, leaves, buds, leaves with stems, and flowers without petals of *R. platyacantha* (R1–R5, respectively), were first compared for total phenolics and flavonoids content. Extracts R2-R5 were characterized by the similar content of phenolics (14.53–13.30 mg GAE/g dried weight, dw), whereas extract R1 contained a significantly lower amount of these compounds (8.61 mg GAE/g dw). The content of flavonoids was comparable between extracts and varied from 2.03 to 2.49 µg/g dw (Table 1).

Phenolic compounds, especially flavonoids, are considered the most active group of natural compounds with broad spectrum of health, food, and cosmetic applications [34]. As shown by several researchers, phenolic compounds and flavonoids are potent antioxidants; therefore, they may serve as effective scavengers of reactive oxygen species (ROS) [35]. Although low amounts of ROS are important for intracellular signaling, elevated ROS levels may cause DNA, lipid, and protein damage and thus lead to the development of premature skin aging, pigmentation disorders, or skin cancer [36,37].

The antioxidant potential of R1–R5 extracts was first compared using DPPH and ABTS radical scavenging assays. In both methods, the highest and lowest antioxidant activity was detected in *R. platyacantha* extracts with the highest and lowest phenolics content, respectively (Table 1).

**Figure 1:**
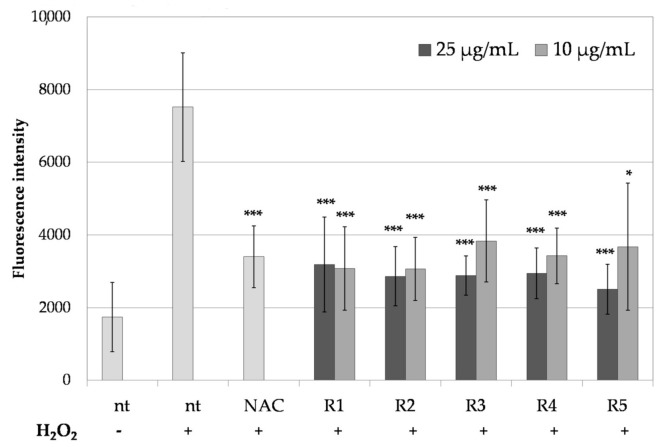
The effect of R1–R5 *R. platyacantha* extracts on the intracellular ROS levels in HaCaT keratinocytes treated for 60 min with 1 mM H_2_O_2_; nt—no pre-treatment, NAC—2 mM N-acetylcysteine; values on graph represent mean ± SD (*n* = 3), *** *p* < 0.001, * *p* < 0.05 in comparison with “nt + H_2_O_2_” sample.

The ability of R1–R5 extracts to protect the cells from the harmful effect of ROS was also analyzed in vitro using spontaneously immortalized human keratinocyte cell line HaCaT [38]. In this assay, the cells were stressed with H_2_O_2_ in the presence or absence of R1–R5 extracts at 25 and 10 µg/mL, and the intracellular ROS generation was monitored using the fluorogenic dye H_2_DCFDA. After the diffusion into the cell, H_2_DCFDA is deacetylated by cellular esterases and subsequently oxidized by ROS into 2′,7′-dichlorofluorescein (DCF) [39]. As shown in Figure 1, stimulation of HaCaT cells with H_2_O_2_ increased the DCF fluorescence intensity around four times, indicating increased intracellular ROS production. Pre-treatment of the cells with R1–R5 extracts reduced the intracellular ROS levels by 2.5 times in comparison with the cells without pre-treatment. Observed reduction of the intracellular oxidative stress was comparable with that of a known ROS scavenger NAC [40].

### 2.2. Chromatographic Analysis of R. platyacantha Extracts

The extracts R1–R5 were subjected for HPLC/ESI-QTOF-MS analysis, and the identified compounds are presented in Table 2. Based on the surface areas of the peaks corresponding to identified compounds (Figure S1), the relative content of phytochemicals was classified as high (+++), moderate (++), or low (+), as indicated in Table 2.

Chromatographic analysis of the extracts obtained from different parts of *R. platyacantha* showed that the most characteristic components are quinic acid (1), methoxygallic acid isomer (11), and methyl brevifolincarboxylate (19). Methoxygallic acid isomer was the most abundant compound in R3 and R5 extracts. The content of quinic acid and methyl brevifolincarboxylate was comparable between extracts R1, R2, R4, and R5 and relatively lower in extract R3.

While quinic acid, ellagic acid, and their derivatives are compounds commonly identified in various *Rosa* sp. extracts [41,42,43], to our knowledge, this is the first report indicating the presence of brevifolin derivatives in a species from *Rosa* genus.

There are very few reports in the literature on the chemical composition of *R. platyacantha* [44,45]. Both publications reported the presence of hydrolysable tannins (gallotannins and ellagitannins) in fruits of *R. platyacantha*. Ellagic and gallic acids were identified in the methanol extract obtained from this plant material [44,45]. The results of the current research confirm the presence of both acids and their derivatives also in extracts obtained from other parts of the *R. platyacantha.* For the first time, the occurrence of brevifolin derivatives were confirmed. The latter compounds are known to occur in pomegranate, sweet oranges, and *Zanthoxylum* species [46]. It is also interesting to note that the pharmacological profile of brevifolin is reported similar to ellagic acid [47]. From the chemical point of view, ellagitannins may also undergo oxidation to compounds containing a dehydrohexahydroxydiphenoyl group, which is also accompanied by the presence of brevifolin carboxylic acid [48]. This compound was also detected in all extracts obtained from *R. platyacantha*.

Among other characteristic components worth mentioning is the presence of gallic acid derivatives as well as flavonoids belonging to flavonols, especially quercetin derivatives. These compounds are known from the scientific literature as effective antioxidants and were previously detected in the extracts from other *Rosa* species [41,42,43,49]. The abundance of quinic acid, quercetin, and gallic acid as well as their derivatives in *R. platyacantha* extracts explains their significant antioxidant activity [50].

### 2.3. Anti-Collagenase and Anti-Elastase Activity of R. platyacantha Extracts

**Figure 2:**
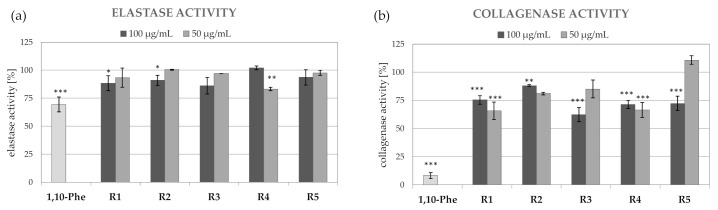
Elastase (**a**) and collagenase (**b**) inhibitory activity of R1–R5 extracts from various parts of *R. platyacantha,* 1,10-phenantroline (1,10-Phe) was used as inhibitor control; values on graphs represent means ± SD (*n* = 3), *** *p* < 0.001, ** *p* < 0.01, * *p* < 0.05.

The upregulated activity of collagenase and elastase plays a pivotal role in wrinkling of the skin via the impairment of collagen and elastic fibers configuration and the subsequent loss of skin elasticity. Increased activity of collagenase and elastase is caused by both intrinsic (chronologic) and extrinsic aging (UV radiation) factors [51,52]. In general, the extracts from *R. platyacantha* were more effective collagenase than elastase inhibitors (Figure 2). All tested extracts showed significant collagenase inhibition at 100 µg/mL, with extract R3 being the most active (38% collagenase inhibition). Extracts R1, R2, and R3 significantly decreased the activity of collagenase also at 50 µg/mL (18–34% inhibition). The activity of elastase was significantly decreased only by R1 and R2 extracts at 100 µg/mL (11–13% inhibition) and extract R4 at 50 µg/mL (16% inhibition).

Several natural inhibitors of collagenase and elastase were identified to date in plant extracts, including *Rosa* species. *R. rugosa* extract was shown to inhibit collagenase activity [53] and *R. damascena* extract from petals inhibits elastase [54]. The extracts from *R. hybrida* and *R. centifolia* showed inhibitory potential toward both collagenase and elastase activities [55,56].

### 2.4. Anti-Tyrosinase Activity of R. platyacantha Extracts

Using plant extracts for the treatment of skin pigmentation disorders gained popularity in recent years [57]. Tyrosinase (EC. 1.14.18.1), a key enzyme of melanogeneis is the most popular target for skin lightening cosmetic ingredients. Tyrosinase catalyzes the rate-limiting conversion of L-tyrosine to L-dihydroxyphenylalanine (L-DOPA) (monophenolase activity) and subsequently to dopaquinone (diphenolase activity) [58].

**Figure 3:**
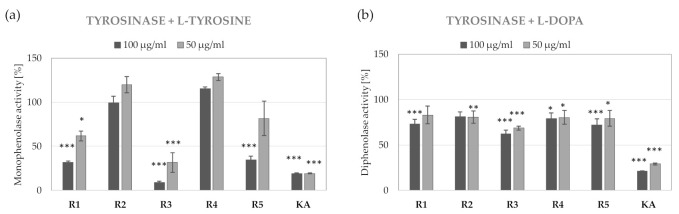
Inhibition of monophenolase (**a**) and diphenolase (**b**) activity of tyrosinase by R1–R5 extracts from various parts of *R. platyacantha;* KA- kojic acid, values on graphs represent mean ± SD (*n* = 3), *** *p* < 0.001, ** *p* < 0.01, * *p* < 0.05.

*R. platyacantha* extracts inhibited monophenolase and diphenolase activity of tyrosinase in a dose-dependent manner, with R3 extract being the most active. At 100 µg/mL, R3 extract inhibits 90% of monophenolase (Figure 3a) and 38% of diphenolase (Figure 3b) activity of tyrosinase. Extracts R2 and R4 decreased diphenolase activity by 20% (Figure 3b) but had no impact of monophenolase activity of tyrosinase (Figure 3a).

### 2.5. Anti-Melanoma Activity of R. platyacantha Extracts In Vitro

Several phenolic compounds identified in *R. platyacantha* extract, including ellagic and gallic acids [59,60], kaempferol [61], and quercetin [62] were previously shown to induce apoptosis in melanoma cell lines in vitro. Therefore, extracts rich in these compounds might be used in melanoma chemoprevention, suppressing the initiation, promotion, and progression of cancer cells. The addition of chemopreventing agents in cosmetics may increase their effectiveness due to the repeated application directly on the skin surface [63].

R1–R5 extracts were analyzed for their cytotoxicity in vitro against human (A375, SH-4) and murine (B16-F10) melanoma cell lines and noncancerous human keratinocytes HaCaT (Table 3). Except for the R1 extract, all analyzed extracts were cytotoxic for the melanoma cell lines. The most significant cytotoxicity was observed for R3 and R5 extracts against the A375 cell line (IC_50_ = 97.31 and 72.90 µg/mL, respectively). The cytotoxic effect of these extracts against noncancerous HaCaT cells was 1.9–2.4 times lower (IC_50_ = 187.30 and 174.20 µg/mL, respectively). To our knowledge, this is the first report showing the anti-melanoma activity of *Rosa* spp. extracts. The extracts from *R. canina* were previously proven cytotoxic for colon, cervix, hepatocellular and non-small cell lung carcinoma and leukemia cell lines [13,14]. *R. rugosa* decreased the proliferation of prostate cancer cells [15]. *R. roxburghii* was shown to induced intrinsic apoptosis in esophageal squamous carcinoma, gastric carcinoma, and pulmonary carcinoma cell lines [64].

### 2.6. Fractionation of R3 Extract and Chromatographic Analysis of the Fractions

Among the compared biological activities, extract R3 was characterized by the most significant antioxidant and anti-melanoma properties as well as an exceptional ability to inhibit both mono- and diphenolase activities of tyrosinase. For that reason, extract R3 was separated into nine fractions (A–I) in order to identify active compounds responsible for the observed antioxidant, anti-melanoma, and tyrosinase inhibitory activities. Fractions A and B were excluded from further analysis, as they contained trace amounts of organic compounds and were not dissolving in DMSO. Fractions C–I were first analyzed for their phytochemical composition. The results are presented in Table 4. Based on the peak’s surface areas in corresponding chromatograms (Figure S2), the relative content of each compound was estimated as high (+++), moderate (++), and low (+) and indicated in Table 4. The most abundant compounds in all fractions were gallic acid and its derivatives (methoxygallic acid isomer and methoxygallic acid glucoside isomer). Fractions E–I contained also quercetin and brevifolin derivatives, whereas these compounds were absent in fractions C and D.

### 2.7. Antioxidant and Anti-Melanoma Activities of Fractions C-I Separated from R3 Extract

The antiradical potential of fractions C–I was compared using DPPH and ABTS scavenging assays, revealing that the most active antiradical compounds were present in fraction E. Fraction C was the least active (Table 5).

The comparison of in vitro cytotoxicity of fractions C-I against HaCaT keratinocytes and A375 melanoma cells showed that the compounds present in fractions F-I are not cytotoxic for both tested cell lines. The most significant cytotoxic effect against melanoma cells was detected in fraction D (IC_50_ = 70.30 µg/mL). This fraction was also the most cytotoxic for noncancerous HaCaT cells, but the calculated IC_50_ value (137.60 µg/mL) was about two times higher than for A375 melanoma cells (Table 6). The two compounds identified in fraction D were gallic acid and methoxygallic acid isomer. The relative content of gallic acid was the highest among all analyzed fractions. Gallic acid was previously shown to induce apoptosis in A375.S2 melanoma cells through the upregulation of the proapoptotic proteins such as Bax, downregulation of antiapoptotic proteins such as Bcl-2, and activation of caspase-9 and caspase-3 [60].

### 2.8. Identification of Tyrosinase Inhibitors from E and F Fractions Separated from R3 Extract

**Figure 4:**
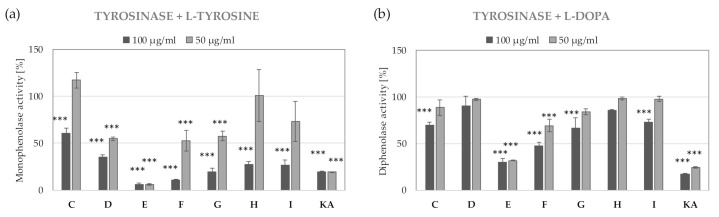
Inhibition of monophenolase (**a**) and diphenolase (**b**) activity of tyrosinase by C-I fractions of closed flower extract (R3) of *R. platyacantha* KA- kojic acid, values on graphs represent mean ± SD (*n* = 3), *** *p* < 0.001.

In the tyrosinase inhibitory studies, fractions E and F showed the most significant inhibition of monophenolase and diphenolase activities. Fraction E reduced the monophenolase activity of tyrosinase by >90% and was more effective than kojic acid at the corresponding concentrations (Figure 4).

**Figure 5:**
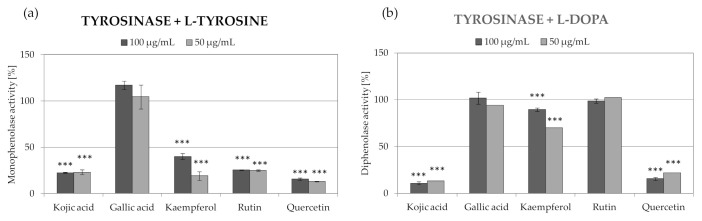
Inhibition of monophenolase (**a**) and diphenolase (**b**) activity of tyrosinase by main constituents identified in fraction E from extract R3, values on graphs represent mean ± SD (*n* = 3), *** *p* < 0.001.

Chromatographic analysis showed that fraction E, characterized by the highest tyrosinase inhibitory activity, contains mostly gallic acid and its derivatives. The content of these compounds in the second most active fraction F was much lower, suggesting that gallic acid and its derivatives are not responsible for the observed tyrosinase inhibitory properties. Other compounds present in E and F fractions included derivatives of kaempferol, quercetin, and rutin. In order to identify the compound responsible for the tyrosinase inhibitory potential of fractions E and F and extract R3, pure reference compounds were analyzed in the same assay. As shown in Figure 5, quercetin, rutin, and kaempferol were effective inhibitors of the monophenolase activity of tyrosinase, which was comparable with the widely used tyrosinase inhibitor—kojic acid. The diphenolase activity of tyrosinase was decreased by quercetin (78% inhibition at 50 µg/mL) and kaempferol (30% inhibition at 50 µg/mL). The mono- and diphenolase activities of tyrosinase were not affected by gallic acid. Based on obtained data, it might be concluded that quercetin, kaempferol, rutin, and their derivatives are responsible for the tyrosinase inhibitory properties of *R. platyacantha* flower buds extract. Quercetin has been already described in a B16 murine melanoma model as an effective tyrosinase inhibitor from *R. canina*. The inhibition of melanogenesis by quercetin was due to the inhibition of both tyrosinase activity and of the protein expression [49].

## 3. Materials and Methods

### 3.1. Chemicals and Reagents

A375 (ATCC CRL-1619) human malignant melanoma, SH-4 (ATCC CRL-7724) human melanoma, and B16-F10 (ATCC CRL-6475) murine melanoma cell lines were purchased from LGC Standards (Łomianki, Poland). Immortalized human keratinocytes HaCaT were purchased from CLS Cell Lines Service GmbH (Eppelheim, Germany). Fetal bovine serum (FBS) was obtained from Pan-Biotech (Aidenbach, Germany). Dulbecco’s modified Eagle’s medium (DMEM)/high glucose, with and without phenol red, Dulbecco’s phosphate buffered saline (DPBS), mushroom tyrosinase from *Agaricus bisporus*, L-tyrosine, 3,4-dihydroxy-l-phenylalanine (L-DOPA), 2,2-Diphenyl-1-picrylhydrazyl (DPPH), 2′,7′-dichlorofluorescin diacetate (H_2_DCFDA), *N*-acetylcysteine (NAC), *N*-Succinyl-Ala-Ala-Ala-p-nitroanilide (SANA), kojic acid (≥99.0%), chlorogenic acid (≥95%), ellagic acid (≥95%), gallic acid (97.5–102.5%), kaempferol (≥97.0%), quercetin (≥95.0%), rutin (≥94.0%), 1,10-phenantroline (≥99.0%), and neutral red solution (3.3 g/L) were purchased from Merck (Darmstadt, Germany). Water (H_2_O), formic acid (HCOOH), and acetonitrile (CH₃CN) for HPLC analysis were purchased from J.T. Baker (Witko, Łódź, Poland).

### 3.2. Plant Material

*Rosa platyacantha* Schrenk plant was collected on 23 of May 2019 in the mountains of Trans-Ili Alatau, Northern Tian Shan mountain region (Almaty, Kazakhstan). Identification of the plant was made by the Institute of Botany and Phytointroduction located in Almaty, Kazakhstan. Collected plant material was dried in room temperature with relative humidity 50 ± 5% in a ventilated premises for a duration of 5 days. A voucher specimen of the plant is being kept in Almaty, in the Institute of Botany and Phytointroduction of the Committee for Science of the Ministry of Education and Science of Republic of Kazakhstan.

### 3.3. Extraction Procedure and Fractionation

For the purpose of this study, five extracts from various parts of *R.* *platyacantha* were prepared, as described in Table 7.

First, 30 g of dried plant material was mixed with 400 mL of 70% methanol and put for 30 min to an ultrasonic bath (Bandelin SONOREX Digital 10P) at 30 °C. Then, the extract was filtered, and a fresh portion of solvent was added (300 mL). After 30 min of ultrasound extraction, the plant material was left for overnight maceration; then, the extract was filtered, a new portion of solvent was added, and ultrasound-assisted extraction was performed. Filtrates from each extraction step were collected, and solvent was removed by rotary evaporator under reduced pressure.

Five mg of extract obtained from *R. platyacantha* closed flowers (R3) was absorbed on small portion of silica gel (230–400 mesh). Dry Column Vacuum Chromatography (DCVC) was used for the fractionation of this extract. Adsorbed extract was loaded in a dry column filled with silica gel (230–400 mesh) and eluted with 200 mL of n-hexane-EtOAc (7:3, *v*/*v*), n-hexane-EtOAc (1:1, *v*/*v*), n-hexane-EtOAc (3:7, *v*/*v*), EtOAc, EtOAc-MeOH (7:3, *v*/*v*), EtOAc-MeOH (1:1, *v*/*v*), EtOAc-MeOH (3:7, *v*/*v*), MeOH, and MeOH-water (9:1, *v*/*v*) successively. Collected fractions were monitored by TLC using n-hexane-EtOAc (1:1, *v*/*v*) and EtOAc-MeOH (1:1, *v*/*v*) as mobile phases. This procedure give nine fractions (A–I).

### 3.4. Total Phenolics and Flavonoids Content

The content of total phenolic compounds was determined as described by Fukumoto and Mazza [65] with slight modifications. First, 150 µL of extracts (1 mg/mL) was mixed with 750 µL of Folin–Ciocalteu reagent (1:10 *v*/*v*, in water) and incubated for 5 min at room temperature. The samples were mixed with 600 µL 7.5% (*m*/*v*) Na_2_CO_3_ and incubated for 30 min at room temperature (RT) in darkness. The absorbance was measured at λ = 740 nm using a DR 600 Spectrophotometer (Hach Lange, Wrocław, Poland). The calibration curve (y = 0.4046x − 0.429; R^2^ = 0.9978) was prepared using 0–100 µg/mL gallic acid. The content of total phenolics was calculated as gallic acid equivalents (GAE) in mg per g of dried extract weight (dw).

The content of flavonoids was analyzed according to the method described by Matejić et al. [66] with some modifications. First, 150 µL of analyzed extracts (1 mg/mL) were mixed with 650 µL reaction mixture (61.5 mL 80% C_2_H_5_OH + 1.5 mL 10% Al(NO_3_)_3_·9H_2_O + 1.5 mL 1 M CH_3_COOK). Following 40 min incubation at RT, in darkness, the absorbance of the samples was measured at λ = 415 nm. The calibration curve (y = 0.313x − 0.3127, R^2^ = 0.9988) was prepared using 0–100 µg/mL quercetin. The content of flavonoids is expressed as quercetin equivalents (QuE) per gram of dried extract weight (dw).

### 3.5. Chromatographic Analysis

The purified samples were analyzed qualitatively by an HPLC/ESI-QTOF-MS system in negative ion mode with the use of a 6530B Accurate-mass-QTOF-MS (Agilent Technologies, Inc., Santa Clara, CA, USA) mass spectrometer with an ESI-Jet Stream ion source. The Agilent 1260 chromatograph was equipped with a DAD detector, autosampler, binary gradient pump, and column oven. The column used as stationary phase was Gemini^®^ 3 µm NX-C18 110 Å, LC Column 100 × 2 mm. Gradient of solvents: water with 0.1% formic acid (solvent A) and acetonitrile with 0.1% formic acid (solvent B) were used as the mobile phases. The following gradient procedure was adopted: 0–45 min, 0–60% of B; 45–46 min, 60–90% B; 46–50 min 90% (B), the post time was 10 min. The total time of analysis was 60 min, with a stable flow rate at 0.200 mL/min. Injection volume for extracts was 10 μL. ESI-QToF-MS analysis was performed according to the following parameters of the ion source: Dual spray jet stream ESI, positive and negative ion mode, gas (N2) flow rate: 12 L/min., nebulizer pressure: 35 psig, vaporizer temp.: 300 °C; *m*/*z* range 100–1000 mass units, with acquisition Mode Auto MS/MS, collision-induced dissociation (CID): 10 and 40 V with MS scan rate 1 spectrum per s, 2 spectra per cycle, skimmer: 65 V, fragmentor: 140 V and octopole RF Peak: 750 V. Identification of compounds was based on Metlin database (https://metlin.scripps.ed) (accessed on 27 January 2021) and compared with literature data.

### 3.6. DPPH Radical Scavenging Assay

The antiradical activity of *R. platyacantha* extracts and fractions was established using a DPPH radical scavenging assay, according to the modified protocol described by Matejic et al. [66]. First, 100 μL of extracts or fractions (0.0005–1 mg/mL) was mixed with 100 μL of DPPH in working solution (25 mM in 99.9% methanol; A540 ≈ 1). Then, 100 μL of the solvent (methanol) mixed with 100 μL DPPH was used as a control sample. After 20 min incubation at RT in darkness, the absorbance of the samples was measured at λ = 540 nm using a FilterMax F5 microplate reader (Molecular Devices, San Jose, CA, USA). Obtained values of measurements were corrected by the absorbance values of the samples without DPPH. The percentage of DPPH radical scavenging was calculated based on the following equation:

% of DPPH˙ scavenging = [1 − (Abs(S)/Abs(C))] × 100%(1)

where Abs(S)—the corrected absorbance of the sample, Abs(C)—the corrected absorbance of the control sample.

Obtained results were used to calculated IC_50_ values defined as the concentration of dried extract/fraction that is required to scavenge 50% of the DPPH radical activity.

### 3.7. ABTS Radical Scavenging Assay

The antioxidant activity of *R. platyacantha* extracts was compared using ABTS radical scavenging assay [67] with modifications. ABTS working solution was prepared by dissolving 7 mM ABTS in 2.45 mM K_2_S_2_O_8_ in distilled H_2_O (A_405_ ≈ 1). Then, 15 μL of extracts diluted in DMSO in the concentration range from 0.0005 to 1 mg/ mL was mixed with 135 μL ABTS working solution. Then, 15 μL DMSO mixed with 135 μL ABTS served as a control sample. Following 15 min incubation at RT in darkness, the absorbance of the samples was measured at λ = 405 nm using a microplate reader (FilterMax F5 Molecular Devices, USA). The obtained values were corrected by the absorbance value of the sample without ABTS. The percentage of ABTS radical neutralization was calculated based on the following equation:

% of ABTS scavenging = [1 − (Abs(S)/Abs(C))] × 100(2)

where Abs(S)—the corrected absorbance of the extract, Abs(C)—the corrected absorbance of the control sample (ABTS + solvent).

The IC_50_ value was defined as the concentration of dried extract in µg/mL that is required to scavenge 50% of ABTS radical activity.

### 3.8. Tyrosinase Inhibitory Assay

The inhibition of the monophenolase and diphenolase activities of mushroom tyrosinase by *R. platyacantha* extracts and fractions was analyzed as previously described by Wang et al. and Uchida et al., respectively [68,69]. For the monophenolase inhibitory assay, 80 µL of phosphate buffer (100 mM, pH = 6.8) was mixed with 20 µL of the analyzed sample or kojic acid as an inhibitory control (final concentrations: 100 µg/mL, 50 µg/mL and 25 µg/mL). Then, 20 µL mushroom tyrosinase working solution (500 U/mL) was added per sample followed by 10 min pre-incubation at RT. Following the addition of 80 µL, 2 mM L-tyrosine the samples were incubated for 20 min at RT in darkness.

The diphenolase inhibitory assay was performed by mixing 120 µL of phosphate buffer (100 mM, pH = 6.8) with 20 µL of diluted samples or kojic acid as an inhibitory control (final concentrations: 100, 50, and 25 µg/mL) and 20 µL mushroom tyrosinase solution (500 U/mL). The reaction mixture was incubated for 10 min at RT. Following the addition of 40 µL 4 mM L-DOPA, the samples were incubated for a further 20 min at RT in darkness.

In both assays, the formation of dopachrome in the presence or absence of analyzed samples was measured spectrophotometrically at λ = 450 nm using a FilterMax F5 microplate reader (Molecular Devices, San Jose, CA, USA). The values were corrected by the absorbance of the extracts without tyrosinase, L-tyrosine, and L-DOPA. A control sample (100% tyrosinase activity) contained phosphate buffer, tyrosinase, an equal volume of the solvent, and the appropriate dose of each substrate. The activity of tyrosinase was calculated based on the equation:

% of tyrosinase activity = [Abs(S)/Abs(C)] × 100%(3)

where Abs(S)—the absorbance of the sample (extract + tyrosinase + substrate), Abs(C)—the absorbance of the control sample (solvent + tyrosinase + substrate).

### 3.9. Elastase Inhibitory Assay

The inhibition of elastase by *R. platyacantha* extracts was established using the protocol described by Horng and co-workers [70]. First, 100 µL Tris-HCl (0.2 M, pH 6.8), containing 0.15 M NaCl and 0.01 M CaCl_2_ was mixed with 15 µL of sample (100 µg/mL and 50 µg/mL), 0.1 M 1,10-phenantroline (metalloprotease inhibitor) or DMSO (solvent control), and 25 µL elastase working solution (50 µg/mL, 2 U/mL, in 0.1 Tris-HCl, pH 6.8). Following 10 min incubation at room temperature, 20 µL of 2.9 mM SANA was added to each sample, mixed, and incubated for 30 min at 37 °C. The absorbance of the samples at λ = 405 nm was measured using a FilterMax F5 microplate reader (Molecular Devices, San Jose, CA, USA). The obtained values were corrected by the absorbance of the diluted extracts without elastase and SANA. The activity of elastase was calculated based on the equation:

% of elastase activity = [Abs(S)/Abs(C)] × 100%(4)

where Abs(S)—the absorbance of the sample (extract + elastase + SANA) and Abs(C)—the absorbance of the control sample (solvent + elastase + SANA).

### 3.10. Collagenase Inhibitory Assay

The collagenase inhibitory potential of the extracts obtained from various parts of *R. platyacantha* at 100 µg/mL and 50 µg/mL was analyzed using Collagenase Activity Colorimetric Test (Sigma Aldrich, St. Louis, MO, USA). Here, 0.01 M 1,10-phenantroline was used as inhibitory control. The analysis and the calculation of collagenase activity (U/mL) was performed according to the manufacturer’s instructions. Then, obtained values were used to calculate the collagenase activity in comparison with the control sample (100% collagenase activity), using the following equation:

% of collagenase activity = [Act(S)/Act(C)] × 100%(5)

where Act(S)—collagenase activity of the analyzed sample and Act(C)—collagenase activity of the control sample.

### 3.11. In Vitro Cytotoxicity Assay

The cytotoxicity of *R. platyacantha* extracts and fractions was investigated using Neutral Red Uptake Test [71]. A375, SH-4 human melanoma, B16-F10 murine melanoma, and HaCaT human keratinocyte cell lines were maintained in DMEM supplemented with 10% FBS at 37 °C in a humidified atmosphere with 5% CO_2_. For the experimental purpose, 3 × 10^3^ cells were plated per well onto a 96-well plate and grown overnight. Then, the cells were treated with various concentrations of *R. platyacantha* extracts (12.5–400 µg/mL) or an equal volume of DMSO as solvent control. Following 48 h of culture, the cells were incubated for 3 h with 33 µg/mL neutral red solution in DMEM containing 1% FBS, rinsed with DPBS and lysed using acidified ethanol solution (50% *v*/*v* ethanol, 1% *v*/*v* acetic acid). The absorbance of the released neutral red was measured at λ = 540 nm using FilterMax F5 microplate reader (Molecular Devices, San Jose, CA, USA) and corrected by the absorbance at λ = 620 nm. The mean measured value for the lysate from control cells was set as 100% cellular viability and used to calculate the percentage of viable cells following extracts treatment. Obtained values were used to calculated IC_50_, which was defined as the concentration of dried extract/fraction decreasing the viability of each cell line by 50%.

### 3.12. Detection of Intracellular ROS using H_2_DCFDA

The influence of R1–R5 *R. platyacantha* extracts on the intracellular ROS levels in H_2_O_2_-treated HaCaT keratinocytes was measured using 2′,7′-dichlorofluorescin diacetate (H_2_DCFDA) assay described by Wu and Yotnda [72] with some modifications. First, 1 × 10^4^ HaCaT keratinocytes were plated per well onto black-walled, 96-well plates and cultured overnight in DMEM supplemented with 10% FBS. The cells were loaded with 5 µM H_2_DCFDA diluted in serum-free, phenol red-free DMEM at 37 °C and 5% CO_2_ for 30 min, in darkness. Diluted R1–R5 extracts (final concentrations 25 µg/mL and 10 µg/mL) or a known ROS-scavenger *N*-acetyl-l-cysteine (NAC, 2 mM) were pre-mixed in serum-free, phenol red-free DMEM with 1 mM H_2_O_2_ and applied to H_2_DCFDA-loaded cells. Equal volume of the serum-free, phenol-red free DMEM was applied to the control cells. Then, the cells were incubated at 37 °C and 5% CO_2_ in darkness. The fluorescence intensity of the forming 2′,7′-dichlorofluorescein (DCF) was measured following 60 min of incubation using a FilterMax F5 microplate reader (Molecular Devices, San Jose, CA, USA) at maximum excitation and emission spectra of 485 and 535 nm, respectively. Obtained values were corrected by the fluorescence of the appropriately diluted R1–R5 extracts, NAC or serum-free, phenol red-free DMEM (background fluorescence).

### 3.13. Statistical Analysis

All experiments were conducted in at least three replicates. Obtained data were analyzed using GraphPad Prism 7.0 Software (GraphPad Software, San Diego, CA, USA). The statistical significance between results was analyzed using one-way analysis of variance (ANOVA) followed by Tukey’s test. All data are showed as mean ± SD.

## 4. Conclusions

The study is the first complex characterization of the chemical profile and selected biological properties of extracts obtained from various parts of *R. platyacantha* grown in the Almaty region in Kazakhstan. The phytochemical studies confirm the presence of gallic and ellagic acids and their derivatives as the most characteristic components of this *Rosa* species. For the first time, the occurrence of brevifolin derivatives was confirmed in genus *Rosa*, while the presence of flavonoids was confirmed in the investigated *R. platyacantha*.

The presented results also indicate that the extract prepared from closed flowers (buds) (R3) of *R platyacantha* is the richest source of phytocompounds with significant antioxidant potential, as confirmed by standard DPPH and ABTS radical scavenging assays as well as in vitro studies on HaCaT keratinocytes. The R3 extract was also effective against human melanoma cells, showing considerably lower cytotoxicity toward noncancerous skin cells. Moreover, closed flowers extract was effectively inhibiting the monophenolase and diphenolase activities of tyrosinase, suggesting its significant skin-lightening potential. Active compounds of the extracts that might be responsible for the observed activities include especially quercetin and its derivatives, e.g., rutin. Gallic acid, ellagic acid, and kaempferol are also active ingredients.

Based on the biological activity profile, flower buds extract from *R. platyacantha* should be considered as an effective active ingredient of skin lightening, anti-aging, and protecting cosmetics. Further studies involving human skin cell lines and 3D tissue models should be performed in order to provide additional data on the safety and cosmetic effectiveness of the *R. platyacantha* extracts.

## Figures and Tables

**Table 1:** Comparison of the total phenolics and flavonoids content and antiradical activity of R1–R5 extracts from various parts of *R. platyacantha;* each value represents mean ± SD (*n* = 3).

	R1	R2	R3	R4	R5	Vitamin C
Total phenolic (mg GAE/g dw)	8.61 ± 0.18	14.53 ± 0.18	13.30 ± 0.16	14.05 ± 0.28	13.68 ± 0.27	-
Flavonoids (mg QE/g dw)	2.42 ± 0.05	2.45 ± 0.03	2.42 ± 0.05	2.49 ± 0.09	2.03 ± 0.06	-
DPPH Scavenging (IC_50_, µg/mL)	2.77 ± 0.05	1.68 ± 0.25	1.50 ± 0.19	1.59 ± 0.14	1.10 ± 0.34	0.96 ± 0.05
ABTS Scavenging (IC_50_, µg/mL)	16.16 ± 1.26	7.16 ± 0.22	10.83 ± 0.85	9.89 ± 0.83	9.21 ± 0.54	0.97 ± 0.06

**Table 2:** Compounds found in *R. platyacantha* R1–R5 extracts after HPLC/ESI-QTOF-MS analysis in negative ion mode; the relative content of identified compounds was indicated as high (+++), moderate (++), or low (+) based on the peak’s surface area in corresponding chromatograms (Figure S1).

No	Retention Time	Name	Formula	Molecular Ion [M − H]^‘^	Fragmentation Ions	R1	R2	R3	R4	R5
1	1.920	Quinic acid	C_7_H_12_O_6_	191.0581	173.0438; 127.0426; 109.0287	+++	+++	++	+++	+++
2	2.507	Citric acid	C₆H₈O₇	191.0362	173.0119; 111.0086	+	+	+	+	+
3	2.955	Gallic acid glucoside isomer	C_13_H_16_O_10_	331.0683	271.0432; 169.0143; 125.0230	+	+	-	+	+
4	3.396	Homoisocitric acid	C_7_H_10_O_7_	205.0373	173.0207; 155.0111; 111.0145	+	-	-	-	-
5	4.092	Gallic acid	C_7_H_6_O_5_	169.0147	125.0258; 107.0141	+	+	++	+	++
6	4.530	Theogallin	C_14_H_16_O_10_	343.0685	191,0497; 127.0389	+	+++	+	+++	+
7	6.495	Gallic acid glucoside isomer	C_13_H_16_O_10_	331.0659	169.0139; 125.0222	+	+	+	+	+
8	7.826	Gallic acid derivative	C_23_H_19_O_18_	581.0449	313.0490; 169.0104	+	-	+	-	+
9	9.877	Methoxygallic glucoside isomer	C_14_H_18_O_10_	345.0820	183.0275; 124.0151	+	-	+	-	-
10	10.127	Chlorogenic acid	C_16_H_18_O_9_	353.0867	191.0373; 135.0308; 109.0238	-	+	-	++	++
11	11.639	Methoxygallic acid isomer	C_8_H_10_O_6_	183.0453	168.0215; 124.0238	++	+	+++	++	+++
12	13.378	Ellagic acid derivative	C_34_H_26_O_22_	783.0488	300.9905; 275.0111; 249.0302	+	-	+	-	+
13	16.619	Ellagitanin derivative	C_30_H_24_O_25_	785.0836	300.9934; 275.0111; 249.0375; 169.0107	+	+	+	-	-
14	17.214	Ellagitannin derivative	C_34_H_26_O_22_	785.0836	300.9981; 275.0185; 249.0428; 162.0121; 125.0163	+	-	-	-	-
15	17.255	Cryptochlorogenic acid	C_16_H_18_O_9_	353.0496	191.0380; 179.0113; 173.0244; 135.0268	+	+	-	+	-
16	17.559	Strictinin	C_27_H_22_O_18_	633.0723	300.9669; 247.9903; 249.0121; 168.9972; 125.0082	-	+	+	+	+
17	19.218	Brevifolin carboxylic acid	C_13_H_8_O_8_	291.0133	247.0116; 205.0041	++	++	+	+++	+
18	20.860	Brevifolin	C_12_H_7_O_6_	247.0235	201.0169; 190.0258; 173.0207; 145.0278; 135.0421	+	+	+	+	+
19	21.130	Methyl brevifolincarboxylate	C_14_H_10_O_8_	305.0290	273.0069; 245.0075; 217.0119; 189.0166; 161.0237; 145.0269; 133.0273; 117.0349	+++	++	++	+++	++
20	23.021	Quercetin galloylglucoside isomer	C_28_H_24_O_16_	615.1014	463.0872; 300.0281; 271.0297; 255.0269; 169.0140; 151.0020; 124.0151; 107.0091	+	+	+	+	+
21	23.281	Quercetin galloylglucoside isomer	C_28_H_24_O_16_	615.1014	463.0900; 300.0276; 271.0283; 255.0223; 169.0140; 150.9999; 124.0139	+	-	-	-	+
22	23.951	Ellagic acid glucoside	C_20_H_16_O_13_	463.0584	300.9994; 226.9924; 200.0078; 173.0221; 145.0286; 117.0374	-	-	++	+	++
23	23.997	Quercetin 3-O glucoside	C_21_H_20_O_12_	463.0896	300.0254; 271.0243; 255.0273; 179.0005; 151.0029; 135.0105; 108.0183	+	+	+	-	-
24	24.351	Ellagic acid	C_14_H_6_O_8_	301.0003	283.9966; 245.0068; 201.0123; 173.0243; 145.0273; 117.0332	+	-	-	-	-
25	25.490	Quercetin glucuronide	C_21_H_18_O_13_	477.0700	301.0350; 255.0315; 178.9994; 151.0048; 107.0153	+++	+	+	+	+
26	26.172	Digalloylglucoside	C_22_H_12_O_13_	483.0237	301.0079; 285.0134; 270.9967; 228.0068; 173.0232; 144.0312; 117.0321	+	-	+	-	+
27	26.326	Quercetin 7-O-glucoside	C_21_H_20_O_12_	463.0896	300.0280; 257.0421; 179.0005; 151.0045; 107.0183	+	-	+	-	+
28	28.380	Quercetin galloylglucoside isomer	C_28_H_24_O_16_	615.1014	301.0386; 255.0328; 179.0003; 169.0139; 151.0065; 125.0231	+++	+	+	+	+
29	29.197	Rutin	C_27_H_30_O_16_	609.1278	463.0905; 300.0289; 271.0273; 255.0265; 179.0031; 151.0050; 107.0139	++	-	-	-	+
30	31.014	Kaempferol rutinoside	C_27_H_30_O_15_	593.1105	285.0352; 255.0270; 227.0305; 150.9958; 145.0285; 119.0489	+	-	+	+	-
31	34.432	Kaempferol	C_15_H_10_O_6_	285.0388	229.0512; 150.9980; 107.0111	+	-	-	-	-

**Table 3:** Cytotoxicity of R1–R5 extracts from various parts of *R. platyacantha* (IC_50_ in µg/mL).

	R1	R2	R3	R4	R5
HaCaT	>500	180.60	241.40	304.30	293.90
A365	>500	120.40	97.31	199.50	72.90
SH4	>500	149.70	169.00	129.90	142.00
B16F10	>500	226.10	187.30	136.80	174.20

**Table 4:** Comparison of the chemical composition of the fractions C-I obtained from extract R3 after HPLC/ESI-QTOF-MS analysis in negative ion mode; the relative content of identified compounds was indicated as high (+++), moderate (++), and low (+) based on the surface areas of the peaks in corresponding chromatograms (Figure S2).

No	Retention Time	Name	Formula	C	D	E	F	G	H	I
1	1.920	Quinic acid	C_7_H_12_O_6_	-	-	-	-	+	+	+
2	2.507	Citric acid	C₆H₈O₇	-	-	-	-	-	-	-
3	2.955	Gallic acid glucoside isomer	C_13_H_16_O_10_	-	-	-	+	+	-	-
4	3.396	Homoisocitric acid	C_7_H_10_O_7_	-	-	-	-	+	-	-
5	4.092	Gallic acid	C_7_H_6_O_5_	-	+++	++	+	+	+	+
6	4.530	Theogallin	C_14_H_16_O_10_	-	-	-	-	+	+	+
7	6.495	Gallic acid glucoside isomer	C_13_H_16_O_10_	-	-	+	+	-	-	-
8	7.826	Gallic acid derivative	C_23_H_19_O_18_	-	-	-	+	-	-	-
9	9.877	Methoxygallic acid glucoside isomer	C_14_H_18_O_10_	-	-	++	+	-	-	-
10	10.127	Chlorogenic acid	C_16_H_18_O_9_	-	-	-	+	-	-	-
11	11.639	Methoxygallic acid isomer	C_8_H_10_O_6_	+++	+++	++	-	+	+	+
12	13.378	Ellagic acid derivative	C_34_H_26_O_22_	-	-	+	+	-	-	-
13	16.619	Ellagitanin derivative	C_30_H_24_O_25_	-	-	+	-	-	-	-
14	17.214	Ellagitannin derivative	C_34_H_26_O_22_	-	-	-	+	-	+	-
15	17.255	Cryptochlorogenic	C_16_H_18_O_9_	-	-	-	-	-	-	-
16	17.559	Strictinin	C_27_H_22_O_18_	-	-	-	+	-	-	-
17	19.218	Brevifolin carboxylic acid	C_13_H_8_O_8_	-	-	-	+	+	+	+
18	20.860	Brevifolin	C_12_H_7_O_6_	-	-	+	-	+	+	+
19	21.130	Methyl brevifolincarboxylate	C_14_H_10_O_8_	-	-	+	+	+	+	+
20	23.021	Quercetin galloylglucoside isomer	C_28_H_24_O_16_	-	-	+	-	-	-	-
21	23.281	Quercetin galloylglucoside isomer	C_28_H_24_O_16_	-	-	+	-	-	-	-
22	23.951	Ellagic acid glucoside	C_20_H_16_O_13_	-	-	-	-	-	-	-
23	23.997	Quercetin 3-O glucoside	C_21_H_20_O_12_	-	-	+	+	+	+	+
24	24.351	Ellagic acid	C_14_H_6_O_8_	-	-	-	-	-	-	-
25	25.490	Quercetin glucuronide	C_21_H_18_O_13_	-	-	-	+	+	+	+
26	26.172	Digalloylglucoside	C_22_H_12_O_13_	-	-	+	-	-	-	-
27	26.326	Quercetin 7-O-glucoside	C_21_H_20_O_12_	-	-	-	-	-	-	-
28	28.380	Quercetin galloylglucoside isomer	C_28_H_24_O_16_	-	-	+	+	+	-	+
29	29.197	Rutin	C_27_H_30_O_16_	-	-	+	+	-	-	-
30	31.014	Kaempferol rutinoside	C_27_H_30_O_15_	-	-	+	+	-	-	-
31	34.432	Kaempferol	C_15_H_10_O_6_	-	-	-	-	-	-	-

**Table 5:** DPPH and ABTS scavenging activity of C-I fractions of flower buds extract (R3) of *R. platyacantha* (IC_50_, µg/mL ± SD); each value represents mean ± SD (*n* = 3).

	C	D	E	F	G	H	I
DPPH Scavenging	11.99 ± 0.96	2.60 ± 0.10	2.17 ± 0.04	6.86 ± 0.50	3.87 ± 0.26	5.89 ± 0.60	5.14 ± 0.57
ABTS Scavenging	2 510.00 ± 449.81	4.77 ± 0.10	4.30 ± 0.55	13.87 ± 0.05	8.52 ± 0.27	11.14 ± 0.91	10.39 ± 0.19

**Table 6:** Cytotoxicity of C-I fractions of closed flower extract (R3) of *R. platyacantha* (IC_50_, µg/mL).

	C	D	E	F	G	H	I
HaCaT	251.50	137.60	190.70	>500	>500	>500	>500
A375	170.60	70.30	205.80	>500	>500	>500	>500

**Table 7:** Extracts prepared from various parts of *Rosa platyacantha* Schrenk.

Extract Symbol	R1	R2	R3	R4	R5
*R. platyacantha* part	Flowers	Leaves	Closed flowers (buds)	Leaves with stems	Flowers without petals

## Data Availability

Data is contained within the article.

## Supplementary Materials

The following are available online, Figure S1: The TIC chromatogram recorded in the negative ionization modes for the *R. platyacantha* extracts R1–R5., Figure S2: The TIC chromatogram recorded in the negative ionization modes for the fractions obtained from *R. platyacantha* extract R3.

## Sample Availability

Samples of the plant material are available from the authors.
