# Supplementary material for: Rosa platyacantha Schrenk from Kazakhstan—Natural Source of Bioactive Compounds with Cosmetic Significance
Source: Molecules. 2021 Apr 28;26(9):2578. doi: 10.3390/molecules26092578 (PMC8124526; doi:10.3390/molecules26092578)
Supplement: Supplementary file 1 [file molecules-26-02578-s001.zip › molecules-1199310-supplementary.pdf]

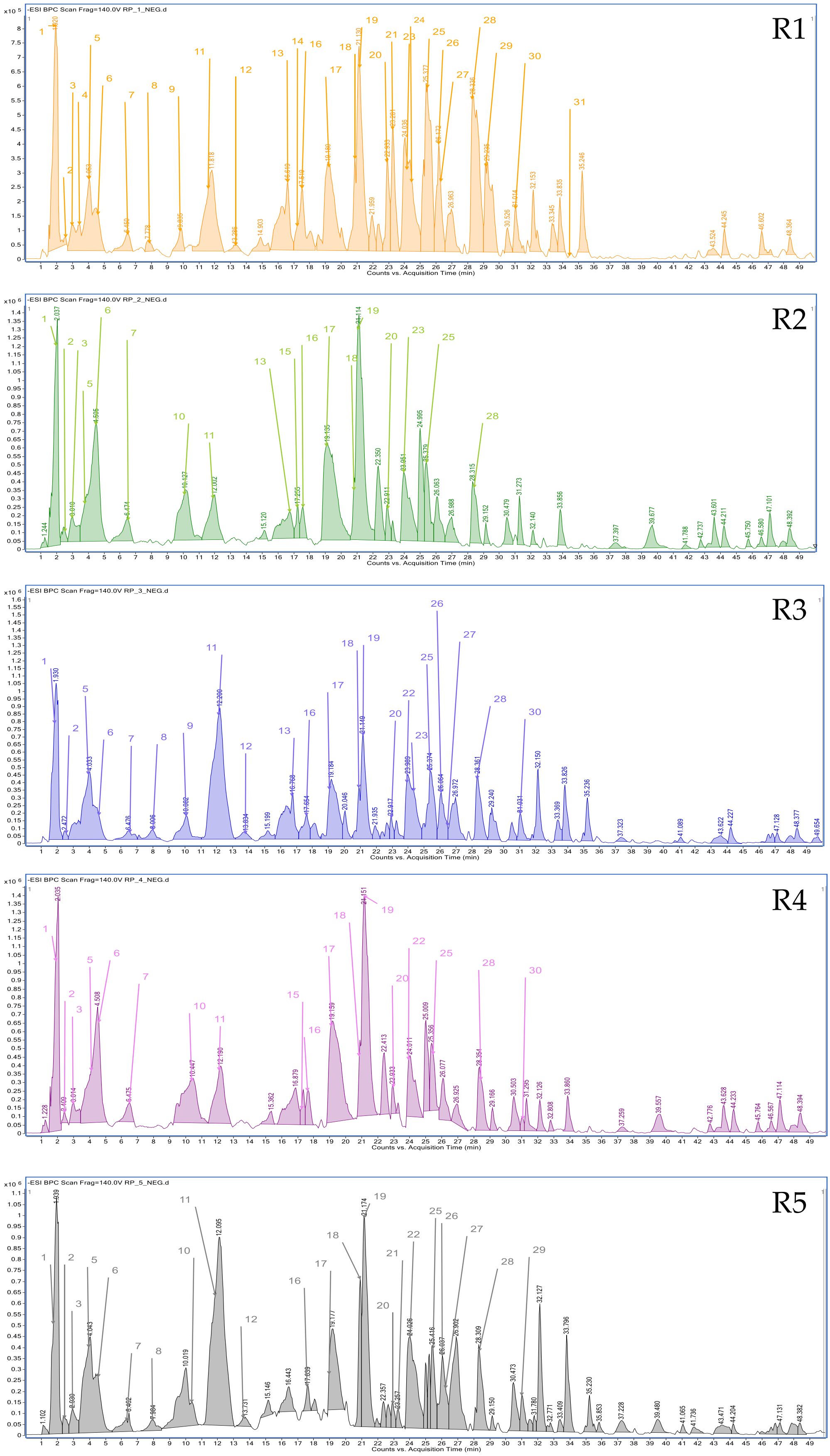

Figure S1. The TIC chromatogram recorded in the negative ionization modes for the *R. platyacantha* extracts R1-R5.

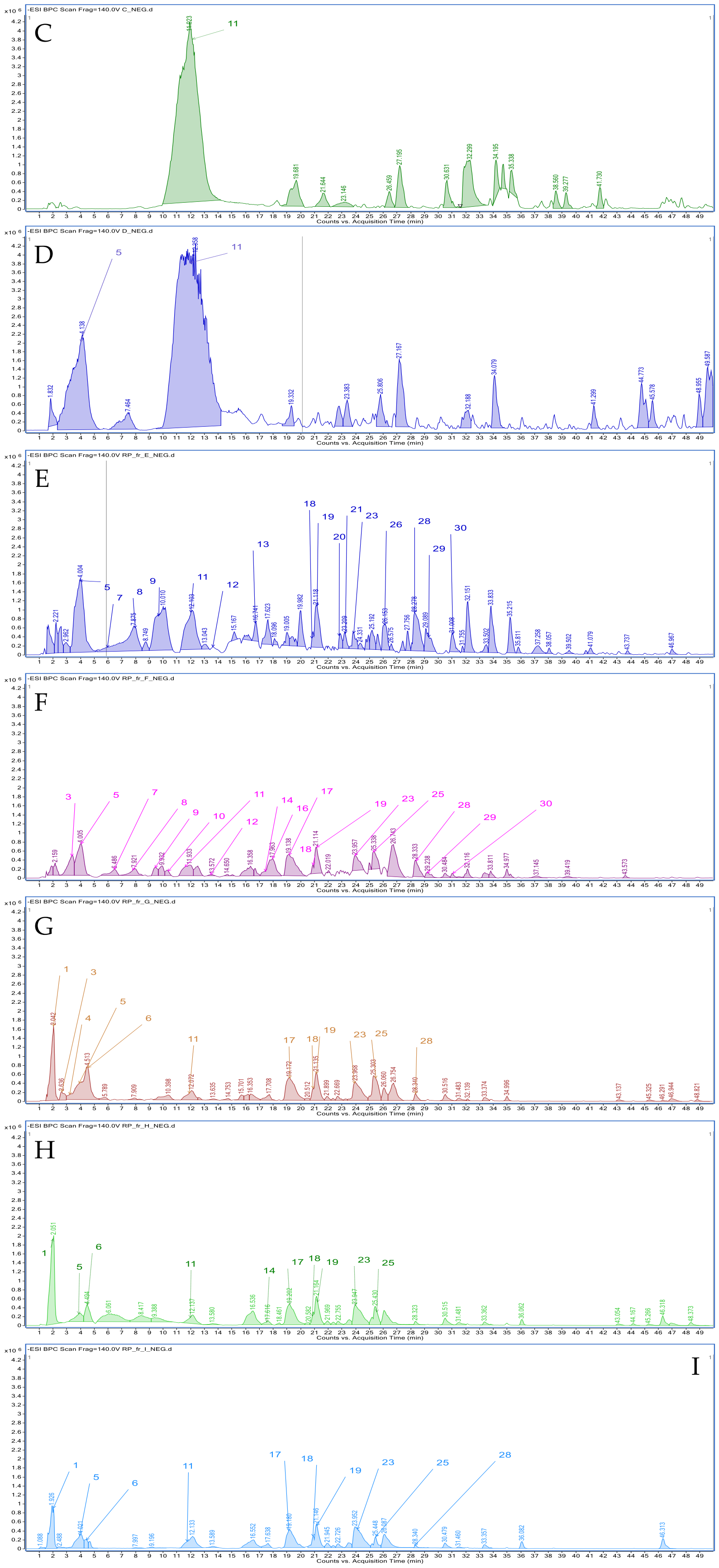

Figure S2. The TIC chromatogram recorded in the negative ionization modes for the fractions obtained from *R. platyacantha* extract R3.
